# Profile, Correlation and Structure of Speed in Youth Elite Soccer Players

**DOI:** 10.2478/hukin-2014-0017

**Published:** 2014-04-09

**Authors:** Malý Tomáš, Zahálka František, Malá Lucia, Teplan Jaroslav

**Affiliations:** 1 Sport Research Centre, Faculty of Physical Education and Sport, Charles University in Prague,.

**Keywords:** testing, performance, sprint, agility, predisposition

## Abstract

Speed, power and agility are important components of fitness and determine the level of success and performance in soccer. The aim of this study was to identify speed variables and to determine their mutual correlation and structure in youth elite soccer players. The research group consisted of players from the Czech U16 national team (n = 22, age = 15.6 ± 0.4 years). Speed variables were assessed using the following tests: a) linear speed: 5 m sprint (S5), 10 m sprint (S10) and 20 m flying sprint (F20); b) the agility: agility test 505 with turning on the dominant (A505D) and non-dominant legs (A505N) and the K-test (K) and c) ball velocity after an instep kick with the dominant (IKD) and non-dominant (IKN) legs. Significant dependence was found for S5 compared with S10, F20 vs. A505N, K vs. A505N (p < 0.01) and S10 vs. F20 (p < 0.05). The factor analysis revealed three components of the latent variable – speed. The first component consisted of linear sprint (S10, S20) and also partially consisted of maximum speed (F20). The second component was primarily composed of agility (A505D, A505N, K) and also included maximum speed (F20). The third independent component represented ball velocity after an instep kick (IKD, IKN). The speed variables in youth elite players exhibited significant heterogeneity from the perspective of performance, as determined by the monitored tests. The structure of the speed predisposition indicated that there were three components of speed. The results of our studies support the notion that each component of speed must be considered independently when designing training programmes.

## Introduction

Speed and explosive power are considered to be prerequisites for the success of youth soccer players (Reilly et al., 2000a). In particular, sprinting at short distances (up to 15 m), vertical jumping and agility have demonstrated a difference in the explosive power between elite and sub-elite youth soccer players (Reilly et al., 2000b). Elite players perform approximately 30 – 40 sprints of various lengths during a match and more than 700 turns (Bloomfield et al., 2007). According to Stolen et al. (2005), high-intensity activities occur approximately every 90 seconds during a match and last for 2 – 4 seconds. The distance that a player covers during sprinting (1.5 – 105 m) indicates that the game requires both acceleration and maximum speed components (Bangsbo, 1994). The analysis of a model of physical activity in a top Italian league (Serie A) indicated that up to 75.8% of high-intensity runs (>19 km·.h^−1^) are performed within 9 m (Vigne et al., 2010). Faude et al. (2012) stated that straight sprints are the most dominant action when scoring goals in professional soccer. Most sprints were conducted without the ball. Thus, straight sprinting should be considered in fitness testing and training. At the level of elite adult players, a player was confirmed to perform approximately 150 – 250 short high-intensity activities during a game, including sprints, which constitute 1 – 11% of the total distance covered by a player during the game; therefore, these high intensity activities place a high demand on a player’s anaerobic capacity (Mohr et al., 2003).

These high-intensity activities appear at irregular intervals and at unequal volume indicators (distance covered) during different types of physical activity (e.g., running sideways, backwards, with and without a ball, accelerating and decelerating). These activities often occur with an incomplete recovery from the previous load. This intermittent and irregular intensity is called the intermittent load of a player.

The ability to maintain and control a body position while quickly changing direction during a series of movements is called agility (Twist and Benicky, 1995). Agility is thus determined by the combination of power, speed, balance and coordination (Draper and Lancaster, 1985). Agility has no general definition but is often described as the ability to quickly change direction, react and stop (Gambetta, 1996). A current study has reported that agility is dependent on two factors: 1) perceptual and decision making factors and 2) factors that are related to the actual mechanics of changing direction (Barnes et al., 2007). Compared with linear speed, a limitation of agility is that the player learns to anticipate the next step (Young et al., 2001).

Together with the performance in tests of acceleration speed and maximum speed, these variables can provide comprehensive information regarding the speed abilities of a player. However, players rarely reach their maximum speed during a match; therefore, the acceleration phase is crucial in game performance (Jovanovic et al., 2011). Acceleration speed, maximum speed and agility in professional soccer players are specific qualities that are relatively unrelated to one another (Little and Williams, 2005). This hypothesis has not been verified in youth elite players. The requirements for playing soccer are multifactorial, and distinguishing characteristics of elite players can be investigated using a multivariate analysis (Reilly et al., 2000b).

The aim of this study was to identify the level of speed variables and to determine their mutual correlation and structure in youth elite soccer players.

## Material and Methods

### Participants

The research group consisted of 22 players of the Czech U16 national team (n = 22, age = 15.6 ± 0.4 years, body height = 177.7 ± 6.9 cm, body mass = 67.9 ± 8.7 kg) who were defined into following positions: 2 goalkeepers, 8 defenders, 7 midfielders and 5 attackers. Twenty players had dominant right legs, whereas 2 players had dominant left legs. The dominant lower limb was determined as the leg that was preferred by players for kicking the ball. The players had 6 training units and one match during a common one-week micro-cycle at their own clubs.

### Data collecting and processing

**Figure 1.**
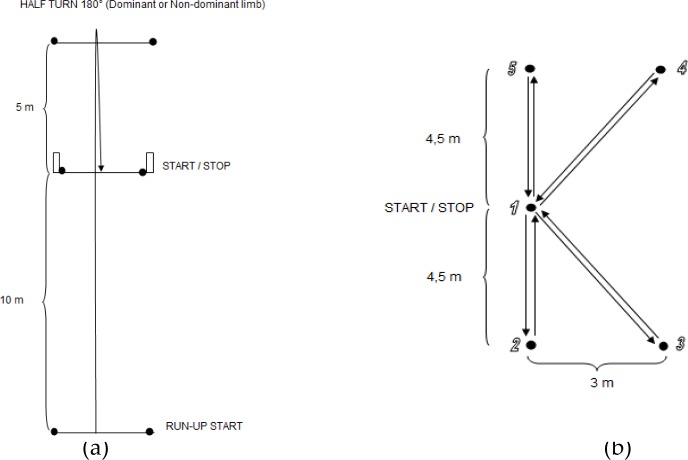
Agility tests used: 505 test (a), K-test (b).

Speed indicators were assessed using field motor tests of running speed. Performance in sprints for 5 m (S5), 10 m (S10) and a flying sprint for 20 m (F20) after a 30 m run-up were measured using photocells (Brower Timing System, Utah, USA). In the sprint speed test, the players ran a 10 m distance; however, the 5 m performance was also measured. This test (S10) was selected to identify the linear speed of the players (Cometti et al., 2001; Little and Williams, 2005). The intragroup correlation coefficient was verified previously with senior soccer players (Mirkov et al., 2008). To assess maximal speed, we used a flying 20 m sprint after a 30 m run-up, which was also used previously with senior professional players (Little and Williams, 2005). The reliability of the maximum speed test with senior soccer players was ICC = 0.93 (Mirkov et al., 2008). To evaluate agility, we used the 505 test (Ellis et al., 2000), which includes both acceleration and deceleration phases of a run with a 180° turn (Figure 1a). A player starts from the “RUN-UP START” position and increases his velocity for 10 m; at the moment when his body crosses the 10 m threshold, the photocells start the clock. The player continues running for 5 m to a line, behind which he turns approximately 180° to the right (left) side and runs back for 5 m. The player performed runs with turns on both the dominant (A505D) and non-dominant limbs (A505N). The reliability of the 505 agility test was previously verified in youth elite Portuguese players, with an intra-group correlation coefficient of ICC = 0.89 (Alves et al., 2010).

**Figure 2.**
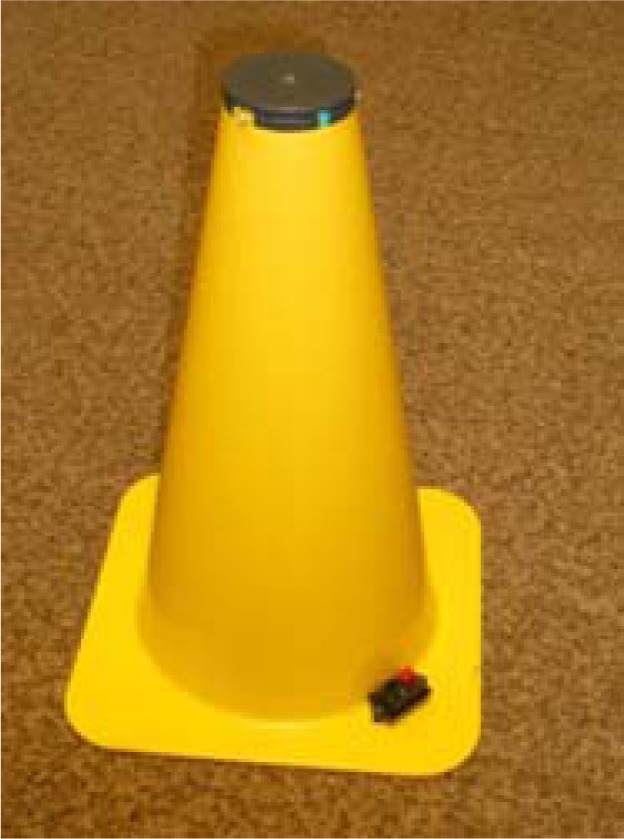
Tested cone with electronic contact switch.

The second agility test that was applied was the K-test (Figure 1b), in which a player runs to cones in a “K” shape at maximum speed. The cones are 35 cm high, and there is a contact switch with a diameter of 7 cm at the top of each cone (Figure 2). The player stands at the middle cone (1), and after starting on his own (pushing the switch), he runs to cone no. 2, where again he taps the switch with his hand and then runs back to the initial cone (no. 1). In this way, he gradually runs to all other cones, and the test ends by pushing the switch at cone no. 1 after returning from cone no. 5.

The players performed 2 attempts in each test; the better performance was used for further processing. The field tests were conducted on artificial grass during a morning training unit at the beginning of the national team camp. Before testing, the players conducted a general warm-up (12 minutes) and a specific speed warm-up (10 minutes) under the direction of the national fitness coach.

The ball velocity after an instep kick was measured using a Stalker ATS radar system (Applied Concepts, Inc., Plano, Texas, USA). We used a stationary radar (operating frequency of 35.1 GHz) that can measure speeds between 0.28 m·s^−1^ and 133.33 m·s^−1^ with an accuracy of ±0.028 m·s^−1^. The radar gun was placed on a tripod at a height of 1.25 m and positioned behind the goal. The radar gun was calibrated immediately before testing according to the manufacturer’s instructions.

The instep kick was performed from the penalty kick mark to the middle of the goal (without a goalkeeper) after a 2 – 3 step run-up; the players were instructed to generate their maximum effort while kicking to achieve the hardest shot. All participants performed 6 attempts (3 × instep kick with the dominant limb [IKD] and 3 × instep kick with the non-dominant limb [IKN]). The rest interval between trials was approximately 3 min, whereas a 10-min interval was provided between dominant and non-dominant kicks. The kicks were performed using a standard competition ball, size no. 5 (mass = 435 g, inflation 700 hPa), with a FIFA (Fédération Internationale de Footbal Association) certificate. The reliability coefficient for velocity measurements using the STALKER ATS radar (repetition method) was r = 0.96 for senior players (Sporis et al., 2007).

Before testing, the players were familiarised with the testing protocol. This study was approved by the ethical committee of the Faculty of Physical Education and Sport, Charles University in Prague, and measurements were performed according to the ethical standards of the Helsinki Declaration.

### Statistical Analysis

To detect the significance among the monitored variables of speed, speed-strength and agility indicators, we used Pearson’s correlation and, subsequently, the coefficient of determination (r^2^). The structure of the speed variables was verified using a factor analysis with the extraction method of principal component analysis with VARIMAX rotation and Kaiser normalisation. The cut-off for the inclusion of a variable in the interpretation of a component was 0.4 (Tabachnik and Fidell, 2006), and all variables were loaded on at least one component. The statistical analysis was performed using the IBM SPSS 19.0 software.

## Results

The mean time of the players over 5 m was 1.09 ± 0.06 s. At the 10 m distance, the players achieved 1.85 ± 0.08 s. In the maximum running speed test (F20), the time was 2.48 ± 0.09 s over a 20 m flying sprint. In the agility test with turning on the dominant and non-dominant legs, the players achieved an identical average time of 2.42 ± 0.09 s, and the players achieved a time of 10.65 ± 0.37 s in the K-test. The velocity of the ball after the instep kick was 102.89 ± 4.45 km·h^−1^ when shooting with the dominant leg and 90.50 ± 7.71 km·h^−1^ when shooting with the non-dominant leg. The correlation among the monitored variables and the coefficients of determination are presented in Table 1.

**Figure 3.**
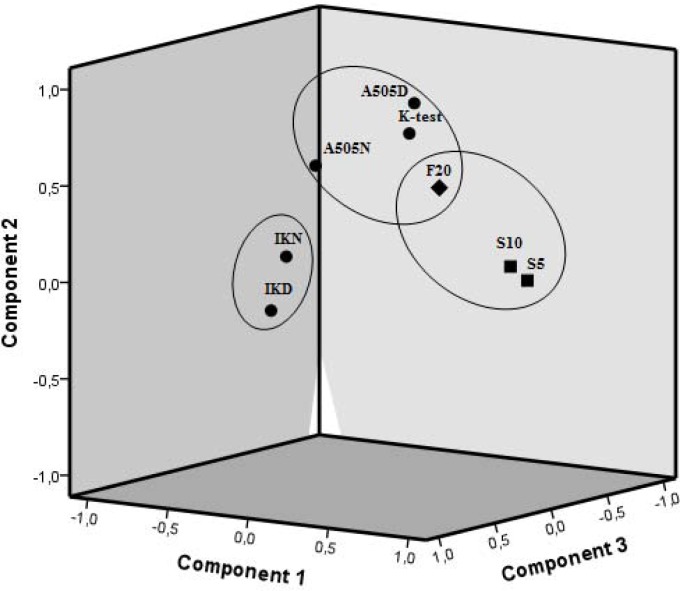
Component plot in rotated space of the monitored speed indicators

The results of the principal components analysis showed three components of the observed latent variable – speed (Table 2, Figure 3). The first component consisted of the linear sprint speed (performance at 5 and 10 m) and also partially consisted of the maximum speed (F20). The second component was primarily composed of agility elements (A505D, A505N, K-test) and also partially composed of the maximum speed (F20). The third independent component represented the level of the ball velocity after the instep kick (IKD, IKN).

## Discussion

### Acceleration

In our research, the players’ time totalled 1.09 ± 0.06 s at the 5 m distance. A similar time (1.09 ± 0.07 s) was also reported (Alves et al., 2010) in youth elite Portuguese players (n = 9, age = 17.4 ± 0.06 years). Superior results (1.07 ± 0.05 s) were reported by Wong and Wong (2009) in youth Chinese national team players (n = 16, age = 16.2 ± 0.06 years) and in elite 16-year-old English soccer players (1.04 ± 0.03 s) (Reilly et al., 2000b).

In the 10 m sprint, the tested players achieved 1.85 ± 0.08 s. Youth players of Rangers FC in the U17 category achieved 1.79 ± 0.03 s at the same distance (McKenna, 2010). The difference between both groups was 3.2%. In the same study, players in the U15 category achieved a superior time (1.81 ± 0.02 s). With respect to senior professional players (n = 106), the time at the 10 m distance was comparable with the results of our tests (1.83 ± 0.08 s) (Little and Williams, 2005). Professional Italian players in the U18 category achieved 1.77 ± 0.06 s in the test at 10 m (Bravo et al., 2008).

High intensity activity during the game is an important element in soccer because increasing speed over a short distance may be necessary not only for adults but also for youth soccer players in crucial phases of the game. For professional players, Andrzejewski et al. (2013) reported that 90% of sprints were performed within 5 seconds. Some authors consider the player’s first steps and the ability to gradually increase their speed as the most important component of the running performance during the game (Dellal et al., 2011; Sleivert and Taingahue, 2004). In terms of these findings, the 5 and 10 m tests appear to be suitable for the assessment of acceleration (Stolen et al., 2005; Strudwick et al., 2002).

### Maximum speed

The players’ time in the maximum running speed test (F20) was 2.48 ± 0.09 s. Elite players of the same age category from a soccer academy in Qatar achieved 2.53 ± 0.11 s (Mendez-Villanueva et al., 2011). In both categories, the Czech elite players were faster (2%). Our players achieved a slower time compared with professional senior players (2.40 ± 0.11 m) (Little and Williams, 2005), with a difference of 3.2%. Maximum speed is determined by the frequency and length of the stride, which negatively affect each other (Coh and Babic, 2010), i.e., an increase in one parameter (running frequency) causes a reduction in the second one (the length of the stride). The length of the stride is, to a certain extent, also determined by anthropometric characteristics (e.g., body height and the length of body segments). The sprint performance of children and adolescents depends on several factors that are mediated by growth and maturation (Malina and Bar-Or, 2004).

Maximum speed is the maximal velocity at which a player can sprint (Little and Williams, 2005). The run-up speed for the development of maximum speed in the game is a player’s action at medium intensity (15 km·h^−1^) (Young et al., 2001). Faude et al. (2012) report that, for professional players, the performance in a straight line sprint is the most important component of the offensive phase when scoring. Most straight line sprints are without an opponent and without a ball.

### Agility

The 505 agility test, compared with the straight line sprint, is characterised by changing directions and greater demands that are placed on players in terms of muscle strength, including a quick deceleration of approximately 20 km·h^−1^ (Chelly and Denis, 2001) and a subsequent maximum reacceleration (Bravo et al., 2008). In the agility 505 test, the players achieved the same time (2.42 ± 0.09 s) when changing direction (turning) with the dominant and non-dominant limbs. Despite identical means, the intra-individual assessment revealed a difference of more than 5% between the dominant and non-dominant limbs in four players. McKenna (2010) reported a better time for both the dominant (right, 2.33 ± 0.05 s) and non-dominant (left, 2.36 ± 0.04 s) limbs in 17-year-old players. In the second agility test (K-test), the average performance of the players was 10.65 ± 0.37 s. The best performance was achieved by a midfielder (10.05 s) and the worst performance was achieved by a goalkeeper (11.43 s), with a difference of 12.1% between these performances. Welsh (1999) stated that not only field players but also goalkeepers must be agile, fast and able to suddenly change direction. Currently in soccer, a player’s running speed is an extremely important skill in terms of an active approach in both offensive and defensive phases, which are characterised by an involvement of a greater number of players. There are fast transfers of groups of players in transition phases of the game from defence to offense and vice-versa, as well as during switching between the phases when the ball is lost (to the defensive phase) or after gaining the ball (to the offensive phase). These actions occur at various large areas with vertical and horizontal circulations of players at a high running speed and fast change of direction.

### Ball velocity after an instep kick

The results of the ball velocity after an instep kick test showed a difference of 12% between the dominant (102.89 ± 4.45 km·h^−1^) and non-dominant (90.50 ± 7.71 km·h^−1^) limbs. The higher values in favour of the preferred leg in elite players were in accordance with the results obtained in previous studies (Dorge et al., 2002; Nunome et al., 2006).

Nunome et al. (2006) found a mean velocity of 115.6 ± 6.1 km·h^−1^ in 5 elite players (age = 16.8 years); these values are 11% higher than those values of our players when shooting with the dominant leg.

### Correlation between variables

Based on the inter-correlation coefficients among the monitored variables, we recorded a significant dependence for the following parameters: S5 vs. S10, p < 0.01; S10 vs. F20 test, p < 0.05; A505N vs. K test, p < 0.01 or vs. F20, p < 0.01; and ball velocity after an instep kick for IKD vs. IKN, p < 0.01. A strong correlation (r = 0.91) was observed for the performance over a short distance (5 or 10 m). Additionally, the high common variability (82%) suggests a common component for performance achievement. Performance in the 10 m sprint test significantly correlated with maximum speed (F20); however, the correlation was lower (r = 0.45) compared with other studies. Specifically, for the correlation between performance in a 10 m sprint test and maximum speed, Sporis et al. (2011) reported that r = 0.68 for elite soccer players from the Serbian U16 national team (n = 25); Mendez-Villanueva et al. (2011) reported that r = 0.26 for U16 elite players (n = 22), and Little and Williams (2005) reported that r = 0.623 in professional adult players (n = 106). Although a correlation does not imply causality between variables, the presented results suggest that acceleration and maximum running speed might be a common factor (general factor). Similar morphological and biochemical determinants of acceleration, maximal speed and agility (i.e., fibre-type proportion) may lead to the assumption that these intercorrelations of the speed qualities are highly related (Little and Williams, 2005). Generally, acceleration is considered to be influenced by the development of concentric forces, impulse and knee extensors activity, whereas maximum speed is related more to the stretch-shortening cycle, lower-limb stiffness and hip extensor activity (Mendez-Villanueva et al., 2011). The authors report differences between acceleration and the maximum running speed from the perspective of age. Dependence between variables in U14 and U18 categories is higher (r = 0.79) than that in the U16 category (r = 0.56). This observation could be explained by the fact that this age range is almost the age at peak height velocity. Differences in maturation in our study are also supported by great variability in the players’ body height and body mass.

Neither agility nor shooting velocity correlated with the sprint performance over a short distance in elite young players. Additionally, no significant correlation between the performance over a short distance and the agility test (zigzag test) was mentioned by Sporis et al. (2011). Performance in the agility test is more demanding on the energy output compared with a straight-line run (Reilly and Bowen, 1984). Greater demands are placed on body control during fast changes in direction and during the effective combining of movements between the cyclic and acyclic characteristics of physical activity. The low level of correlation between these parameters could have been caused by differences between energetic systems that were used during the tests (Alemdaroglu, 2012).

Our results are contrary to the results of Draper and Lancaster (1985) who reported a significant correlation between the 505 agility test and acceleration speed. Little and Williams (2005) found significant correlations between acceleration speed (S10) and maximum speed (F20); acceleration speed and zigzag agility test; and maximum speed and agility in 106 professional senior players. Despite these high correlations, the authors suggested an independent component of velocity, which was based on a low coefficient of determination between these tests (S10 vs. F20, r = 0.62 [39%]; S10 and agility [12%] and F20 and agility, r = 0.35 [21%]). Thomas and Nelson (2001) stated that a common variability of two indicators that are lower than 50% implies that these indicators are specific or independent in their inherent nature.

The 505 agility test with turning on the non-dominant leg significantly correlated with the results of the maximum speed test at 20 m and with the results of the agility K-test. Despite this significant correlation, the coefficient of determination was low, at 0.38 and 0.40, respectively. Regarding stepwise regression, Jones et al. (2009) reported that maximum speed (5 m flying run between 20–25 m distance) explained up to 58% of the variance in the 505 agility test. The next determinant of performance in the 505-agility test was performance in knee flexor eccentric contraction. Although Markovic et al. (2007) reported that leg extensor strength performance was a poor predictor of agility performance, surprisingly, an insignificant correlation was found in A505D vs. A505N performance (p > 0.05). Based on the results of our study, there appears a requirement to verify this insignificant correlation using another agility test with a change of direction on both sides or by identifying the occurrence of strength asymmetries (bilateral strength deficit) between the kicking (dominant) and standing (non-dominant) legs using an isokinetic dynamometer. Despite an identical average performance in these tests, there were significant differences between players from an intra-individual point of view. A significant difference (more than 5%) in performance indicates a “preference” for one of the sides. In terms of implications for training practice, eliminating these differences by individual and specific training is necessary. In terms of laterality and muscle force symmetry, some studies have revealed muscle imbalances in the knee extensors and flexors in elite soccer players (Lehnert et al., 2011; Maly et al., 2010).

Because a so-called relative independence is applied to speed, speed-strength and agility abilities (least generalised), implementing exercise types that respect the metabolic and time-spatial criteria of those physical activities, which the effect of stimulation is desired for, is necessary for their stimulation. These specific exercises, along with optimally arranged adaptation stimuli, should establish a high transfer to a particular movement task that is performed under competitive conditions (specific exercises).

The results of our study indicated that the components of short linear speed and agility were relatively independent in youth elite soccer players, which were in accordance with the results of other studies that have suggested that speed and agility are mutually independent (Ellis et al., 2000). Therefore, speed and agility training should differ concerning the stimulated ability (Draper and Lancaster, 1985; Ellis et al., 2000; Little and Williams, 2005; Young et al., 2001).

In soccer practice, speed abilities are often stimulated not in an isolated way but in a combined way or even specifically (using the ball and modelled game situation or using a principle, such as running behind the defence, tackling an opponent with a countermovement, etc.). Our results showed that, in youth elite soccer players, there are different components of running speed that should be considered when preparing individual training plans by fitness coaches with the aim to improve existing insufficiencies. The results of this study may help with the identification and selection of talented athletes or in the determination of criteria for their selection, respectively. Reilly et al. (2000b) mentioned that talent identification in soccer is more complex than in individual sports, where there are discrete objective measures of performance.

The analysis of speed abilities as a latent variable, which was monitored by selected speed indicators, revealed three components of speed (short linear speed, agility and speed of the kick). The variable, “20 m flying”, shared a common variance with the first and second exploration components. Despite the common feature of speed, which involved exerting a maximum effort in a physical activity, relative independence between some speed parameters was determined. In the intra-individual evaluation of players in the A505 agility test, we noticed remarkable differences in some players between the dominant and non-dominant sides in terms of the task that was performed. These results indicate the requirement for monitoring and subsequent correction of the performance deficit that was found in the speed test from its implementation on both sides. Despite the homogeneity of the group (national team), the results of the study suggested that high variability occurs in youth players in this age category in terms of their performance in speed tests. Because of the small sample size in this study, these results may not be generalized.

However, from the point of view of training practice, distinguishing the components of stimulated speed (short linear speed, maximum speed, agility and acyclic speed) is essential. Despite a similar neurophysiological basis, the results are independent between some variables. Acceleration, maximum running speed agility and kick speed are determined by a combination of specific physiological, metabolic, biomechanical and morphological factors. Therefore, these speed components require different training techniques. Generally, acceleration is influenced by the development of concentric forces, impulse and knee extensors activity, whereas maximum speed is related more to the stretch-shortening cycle, lower-limb stiffness and hip extensor activity (Mendez-Villanueva et al., 2011).

From a practical point of view, identifying the players’ weaknesses (components) based on diagnostics, developing these weaknesses using group and individual training and repeatedly diagnosing these weaknesses are required. Our players achieved poor results in the sprint at 10 m compared with those results that have been reported in the foreign literature. Based on the conducted diagnostics, the fitness coach of the national team designed an individual training plan for each player. A physically well-prepared player, in terms of his speed abilities, can clearly determine his success in the current elite youth soccer international competitions. The results of our studies indicate that each component of speed must be considered independently when designing training programmes.

## Figures and Tables

**Table 1:** Correlation (r) and proportion of common variability (r^2^) between the observed variables

Variable		S5	S10	F20	K	IKD	IKN	A505D	A505N
**1.** S5	*r*	1							
	r*^2^*	1							
**2.** S10	*r*	0.91^**^	1						
	*r^2^*	0.82	1						
**3.** F20	*r*	0.32	0.45^*^	1					
	*r^2^*	0.10	0.20	1					
**4.** K	*r*	0.24	0.39	0.36	1				
	*r^2^*	0.06	0.15	0.13	1				
**5.** IKD	*r*	−0.04	0.12	0.08	0.06	1			
	*r^2^*	0.00	0.01	0.01	0.00	1			
**6.** IKN	*r*	0.05	0.19	0.26	0.21	0.70^**^	1		
	*r^2^*	0.00	0.04	0.07	0.04	0.49	1		
**7.** A505D	*r*	0.01	0.04	−0.01	0.22	0.11	0.20	1	
	*r^2^*	0.00	0.00	0.00	0.05	0.01	0.04	1	
**8.** A505N	*r*	0.24	0.27	0.56^**^	0.63^**^	−0.10	0.18	0.30	1
	*r^2^*	0.06	0.07	0.31	0.40	0.01	0.03	0.09	1

S5 – 5 m sprint; S10 – 10 m sprint; F20 – 20 m flying sprint; K – agility test; IKD – ball velocity after an instep kick with the dominant leg; IKN – ball velocity after an instep kick with the non-dominant leg; A505D – agility 505 test with a turn on the dominant limb; A505N – agility 505 test with a turn on the non-dominant limb;

* - p<0.05;

** - p<0.01.

**Table 2:** Rotated component matrix of the monitored speed indicators

	Component		
	1	2	3
S10	**0.944**	0.148	0122
S5	**0.935**	0.050	−0.042
A505N	0.206	**0.891**	−0.074
K	0.266	**0.759**	0.054
A505D	−0.219	**0.563**	0.198
F20	**0.489**	**0.507**	0.106
IKD	0.021	−0.057	**0.936**
IKN	0.084	0.223	**0.889**

S5 – 5 m sprint; S10 – 10 m sprint; F20 – 20 m flying sprint; K – agility test; IKD – ball velocity after an instep kick with the dominant leg; IKN – ball velocity after an instep kick with the non-dominant leg; A505D – agility 505 test with a turn on the dominant limb; A505N – agility 505 test with a turn on the non-dominant limb
